# Expanding the Knowledge of the Geographic Distribution of *Trypanosoma cruzi* TcII and TcV/TcVI Genotypes in the Brazilian Amazon

**DOI:** 10.1371/journal.pone.0116137

**Published:** 2014-12-31

**Authors:** Valdirene dos Santos Lima, Samanta Cristina das Chagas Xavier, Irene Fabíola Roman Maldonado, André Luiz Rodrigues Roque, Ana Carolina Paulo Vicente, Ana Maria Jansen

**Affiliations:** 1 Laboratório de Biologia de Tripanosomatídeos, Fundação Oswaldo Cruz, FIOCRUZ, Rio de Janeiro, Rio de Janeiro, Brazil; 2 Laboratório de Genética Molecular de Microorganismos, Oswaldo Cruz, FIOCRUZ, Rio de Janeiro, Rio de Janeiro, Brazil; Centro de Pesquisa Rene Rachou/Fundação Oswaldo Cruz (Fiocruz-Minas), Brazil

## Abstract

*Trypanosoma cruzi* infection is a complex sylvatic enzooty involving a wide range of animal species. Six discrete typing units (DTUs) of *T. cruzi*, named TcI to TcVI, are currently recognized. One unanswered question concerning the epidemiology of *T. cruzi* is the distribution pattern of TcII and hybrid DTUs in nature, including their virtual absence in the Brazilian Amazon, the current endemic area of Chagas disease in Brazil. Herein, we characterized biological samples that were collected in previous epizootiological studies carried out in the Amazon Basin in Brazil. We performed *T. cruzi* genotyping using four polymorphic genes to identify *T. cruzi* DTUs: mini-exon, 1f8, histone 3 and gp72. This analysis was conducted in the following biological samples: (i) two *T. cruzi* isolates obtained by culturing of stools from the triatomine species *Rhodnius picttipes* and (ii) five serum samples from dogs in which trypomastigotes were observed during fresh blood examination. We report for the first time the presence of TcII and hybrid DTUs (TcV/TcVI) in the Amazon region in mixed infections with TcI. Furthermore, sequencing of the constitutive gene, gp72, demonstrated diversity in TcII even within the same forest fragment. These data show that TcII is distributed in the five main Brazilian biomes and is likely more prevalent than currently described. It is very probable that there is no biological or ecological barrier to the transmission and establishment of any DTU in any biome in Brazil.

## Introduction

Trypanosomiasis by *Trypanosoma cruzi* is primarily an ancient sylvatic enzooty involving a wide range of mammalian species and triatomine vectors in the Americas. Humans were likely included in the transmission cycle as soon as they arrived in the Americas approximately 20,000 years before present (bp) [1]. Since the discovery of the parasite and its cycle by Carlos Chagas (1909), the high morphologic, biologic, biochemical, and more recently molecular variability of *T. cruzi* isolates, has been observed and discussed [2]. The currently employed molecular tools allow for the recognition of six discrete typing units (DTUs), named TcI to TcVI [3]. Nevertheless, the complexity of *T. cruzi* remains unresolved, considering, for example, the recent discovery of an additional *T. cruzi* genotype in bats [4] and the recognition of heterogeneity within TcI [5].

*T. cruzi* is diploid, genetically very polymorphic, and has a clonal structure that manifests a lack of (or very restricted) sexuality [6]–[7]. Nevertheless, a large body of evidence points to the importance of hybridization events as the cause of the extensive heterogeneity of the taxon [8]. The two more divergent lineages are TcI and TcII, whose separation time is still under debate, as it ranges from 3 to 88 million years bp [9], [10]. The more recently diverged DTUs are TcV and TcVI, which resulted from at least one hybridization event that is estimated to have occurred 0.9 million years bp [11].

The epidemiology of the *T. cruzi* DTUs remains a challenging subject. Large gaps in knowledge concerning the distribution of the distinct DTUs are due mainly to difficulties obtaining representative samples of such a widely distributed enzootic taxon as *T. cruzi*. Currently, TcI has been reported as the most frequently isolated of all mammalian taxa throughout the geographic range of the parasite in an ample variety of biomes and habitats [3]. In comparison, DTUs III and IV are isolated less frequently, although they are also widely distributed [3], [12]–[14]. Very little is known about the wild hosts of the hybrid DTUs V and VI, which until now, have been primarily isolated from humans or from domiciliated triatomines [3], [15]–[16]. In Brazil, there is one single report of TcV infecting a wild host, the caviomorph rodent species *Thrichomys laurentius* [17].

The sylvatic hosts and distribution of TcII in the wild also need to be clarified. This parental and ancient *T. cruzi* DTU, which was previously associated primarily with human infection, has been reported to occur in the central belt of South America, which covers the countries of Brazil, Chile, Colombia, Bolivia, Uruguay and Paraguay [3], [15]–[16]. Above the Amazon region, TcII was found to infect *Triatoma dimidiata* in Guatemala [16]. In Brazil, this DTU has been found to infect a broad range of mammalian species in the Atlantic Forest, Caatinga, Pantanal and Savannah biomes [13], [18]–[21]. Despite its ability to infect a large variety of wild host species, TcII has been isolated from a smaller number of animals, and therefore, it has been proposed that this DTU occurs in more focal cycles [19]. In recent decades, *T. cruzi* isolates from the Amazon region have been subjected to several studies involving molecular characterization. However, only the TcI, TcIII and TcIV DTUs have been reported in this biome [22]–[24]. Altogether, the emergence of Chagas disease in the Amazon region and the generally sparse knowledge about this biome led us to study the enzootic transmission cycle of *T. cruzi* since 2006 in this region [25]–[27]. Herein, we extend these studies and provide new data on the distribution of Tc hybrids and TcII among triatomines and dogs.

## Materials and Methods

### Study area

**Figure 1:**
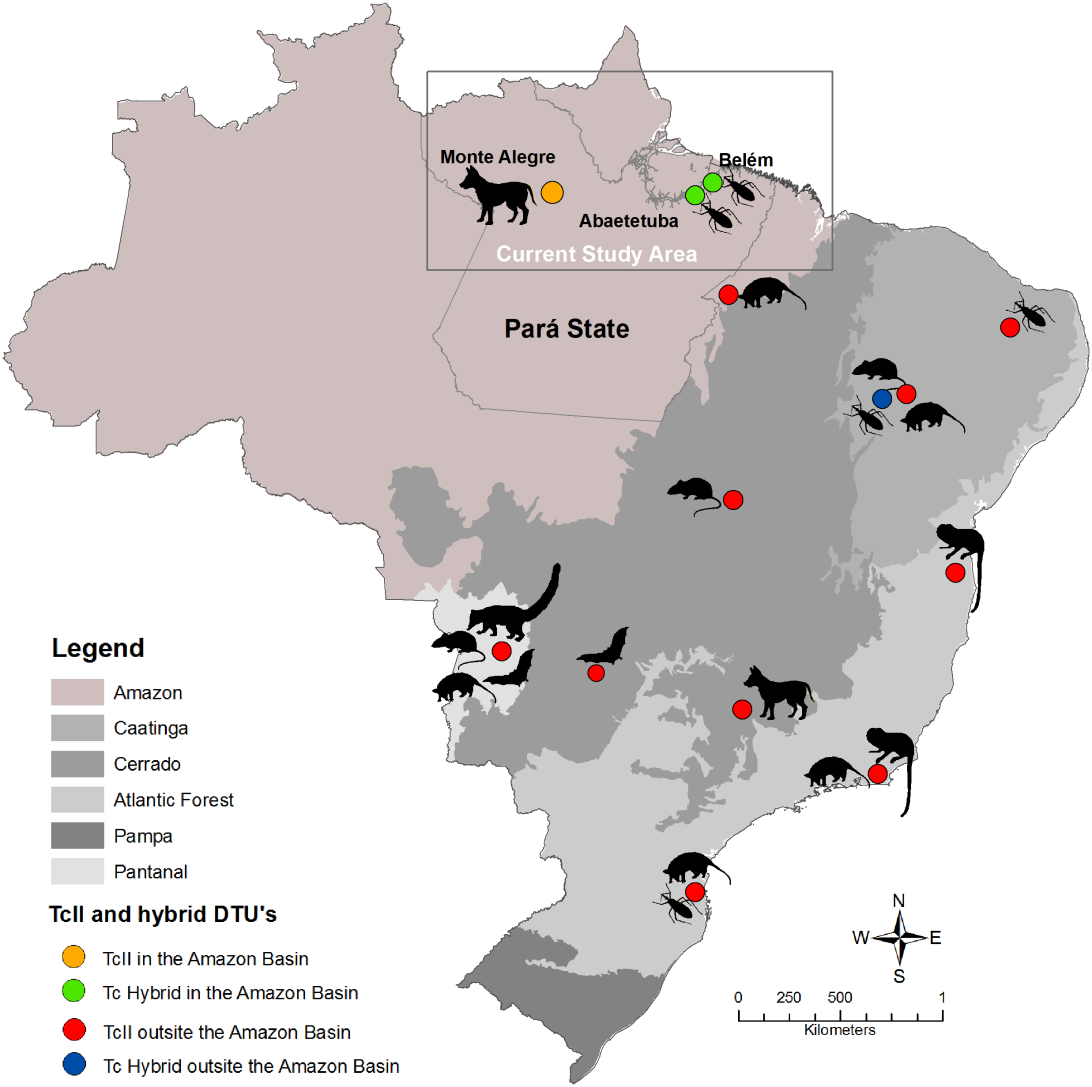
The distribution of *Trypanosoma cruzi* TcII and hybrid genotypes in the Amazon (present study) and in Brazil (published data). Red circles indicate the location of TcII isolates, blue circles indicate the localization of hybrid isolates, and black symbols represent mammalian and vector host species. The upper right figure shows the localization of TcII and hybrid DTUs from this study in the state of Pará. Footnotes: Atlantic Forest biome [19], [37]–[38]; Caatinga biome [17], [20]; Cerrado biome [21]; Pantanal biome [13].

This study was conducted in three municipalities/localities in the state of Pará. These were Abaetetuba/Ajuaí (01°43′24″S; 48°52′54″W) and Belém/Val-de-Cans (01°27′21″S; 48°30′16″W) in the northeastern mesoregion of the state and Monte Alegre/Setor 11 (01°38′20″S; 54°14′32″W) in the lower Amazon mesoregion of the state [26]–[27]. The common climate is characterized as tropical humid with regular rainfall and winds and temperatures between 27°C and 36°C. In most of the collection areas, the original native vegetation (Amazonian forest) is being replaced by an extensive açaí fruit monoculture, with a few remaining patches of the original vegetation at river banks (Fig. 1). No specific permissions were required for these locations.

### Biological samples

For this study of the ecology of *T. cruzi* DTUs in the Amazon region, dogs (N = 422) and triatomine insects (N = 495) were examined [25]–[27]. *T. cruzi* isolates derived from two *Rhodnius pictipes* and five serum samples of dogs with patent parasitemia (positive blood slide smears) from rather distant regions inside Pará were characterized. The two *T. cruzi* isolates obtained from *R. pictipes* were captured in palm trees (*Attalea phalerata*) in Rio Ajuaí (LBT 1458), Abaetetuba municipality, and Val de Cans (LBT 1814) localities (Table 1). *T. cruzi* isolates were obtained by culturing the intestinal contends of the triatomine in NNN+LIT biphasic medium supplemented with 10% fetal bovine serum. When cultures of the two isolates reached the exponential phase of growth, they were subjected to DNA extraction using the phenol-chloroform method, as described elsewhere [28], and deposited in the Coleção de *Trypanosoma* de Mamíferos Silvestres, Domésticos e Vetores, Fiocruz – COLTRYP (COLTRYP 00458 and COLTRYP 00060, respectively).

**Table 1:** *Trypanosoma cruzi* molecular characterization in naturally infected hosts from the state of Pará, Brazil.

*T. cruzi* samples(biological material)	Hostspecies	Municipality	Mini-exon assay^b^	OtherGenotypingprotocols	*T. cruzi*characterization
LBT 1458 (isolate)	*Rhodnius pictipes*	Abaetetuba	TcI and TcII/V/VI	-	TcI and TcII
LBT 1458 (clone 5)		Abaetetuba	TcII-V-VI	1f8 gene^c^	TcII
LBT 1458 (clone 7)		Abaetetuba	TcII-V-VI	1f8 gene^c^	TcII
LBT 1814 (isolate)	*Rhodnius pictipes*	Belém	TcI and TcII/V/VI	Histone 3 gene^d^	TcI and TcII
LBT 1818^a^ (DNA)	*Canis familiaris*	Monte Alegre	TcI	-	TcI
LBT 1819^a^ (DNA)	*Canis familiaris*	Monte Alegre	TcI and TcII/V/VI	-	TcI and TcII/TcV/TcVI
LBT 1820^a^ (DNA)	*Canis familiaris*	Monte Alegre	TcI	-	TcI
LBT 1821^a^ (DNA)	*Canis familiaris*	Monte Alegre	TcI	-	TcI
LBT 1822^a^ (DNA)	*Canis familiaris*	Monte Alegre	TcI and TcII/V/VI	Histone 3 gene^d^ and mini-exon sequencing^e^	TcI and TcV/TcVI

(-) Not available;

a *Trypanosoma cruzi* kDNA positive serum samples from Xavier et al. [27];

b Mini-exon assay according to Fernandes et al. [29];

c PCR-RFLP assay of the 1f8 gene according to Rozas et al. [31];

d PCR-RFLP assay of the histone 3 gene according to Westenberger et al. [32];

e Sequence of a 250 bp fragment from the mini-exon gene. GenBank accession number KJ402456.

**Figure 2:**
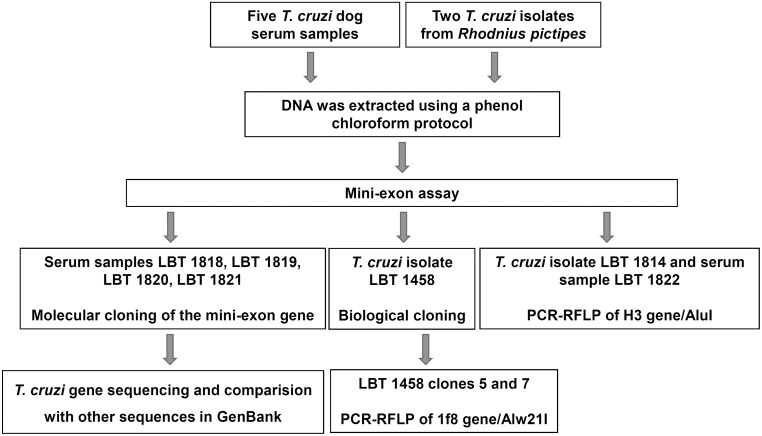
Schematic representation of the methodology used for *Trypanosoma cruzi* typing of distinct biological samples. Serum and feces derived respectively from five naturally infected dogs and axenic cultures of feces obtained from two *Rhodnius pictipes*.

The owners of the dogs from which we obtained the serum samples were from a rural locality in the Monte Alegre municipality (Table 1). The scarce population of this area lives off of subsistence agriculture and uses dogs for hunting. Notably, the dogs were autochthonous and never moved to other sites. Canine infections were detected by fresh blood smear examination and confirmed as *T. cruzi* by PCR of the variable region of the kDNA [27]. Unfortunately, we did not have the opportunity to perform hemocultures from these dogs. The DNA from the five canine serum samples, LBT 1818, LBT 1819, LBT 1820, LBT 1821 and LBT 1822 was extracted using the phenol-chloroform method, as for parasite culture extraction, except without SDS in the pre-treatment (Fig. 2).

### Mini-exon assay and cloning of mixed *T. cruzi* DTUs samples

All *T. cruzi* samples were initially subjected to multiplex PCR of the mini-exon gene performed as described by Fernandes [29], to identify three *T. cruzi* DTU groups and *T. rangeli*: TcI (200 basepairs-bp), Tc2 (TcII/TcV/TcVI–250 bp), zymodeme 3 (TcIII/TcIV–150 bp) and *T. rangeli* (100 bp) (Fig. 2).

The LBT 1458 isolate presented controversial results in the mini-exon assay when samples from a new extraction were typed using the same method and conditions. Therefore, to assess the true subpopulation composition of this isolate, biological cloning was performed. For this purpose, we used solid phase medium tecnique in a Petri dish [30] and selected two Tc2 clones (Fig. 2).

PCR products from canine serum samples (LBT 1819 and LBT 1822) that showed mixed *T. cruzi* DTUs by mini-exon assay were cloned using the pGEM-T Easy Vector System (Promega, Madison, WI, USA) following the manufacturer’s protocol. Each colony grown corresponded to one individual clone containing an insert (amplicon) of both DTUs. The colonies (clones) were randomly collected and subjected to PCR of the mini-exon gene [29] to select clones with 250 bp fragments corresponding to genotypes TcII, TcV or TcVI.

### PCR-RFLP for *T. cruzi* DTU characterization

The following protocols for genotyping the mixed samples were performed depending on the nature of the biological samples: (a) to characterize LBT1458 clones, we performed restriction fragment length polymorphism (RFLP) analysis of the nuclear 1f8 gene after digestion with the Alw21I enzyme, which distinguishes DTUs TcII from TcV and TcVI [31] and (b) PCR-RFLP of the gp72 gene and analysis of fragments digested with the TaqI enzyme were performed as a confirmatory marker to discriminate TcII and to distinguish TcV from TcVI [31] (Fig. 2).

For genotyping the original *T. cruzi* isolate (LBT 1814) and canine serum sample LBT 1822, we used PCR-RFLP of the histone H3 gene and the digestion with the AluI enzyme because this marker distinguishes TcII from TcV and TcVI without overlapping fragments (TcV and TcVI) [21], [32]. Each reaction included negative and positive controls from representative DTUs. The PCR and RFLP results were visualized in 3% agarose gels stained with ethidium bromide under UV illumination (Fig. 2).

### *T. cruzi* gene sequencing of mini-exon and gp 72 genes

#### Mini-exon gene

To compare the mini exon gene with other sequences in GenBank and to identify the *T. cruzi* DTU, we sequenced a 250 bp fragment of the mini-exon of sample LBT 1822. This fragment was obtained after molecular cloning of this gene. The primers used were Tc2 (TcII/TcV/TcVI) and Exon, the same primers that were used for the amplification of this fragment in the multiplex assay.

#### Glycoprotein 72 gene (gp72 gene)

To clarify the unusual profile exhibited by the RFLP protocol for gp72 in LBT 1458 cl 5 and cl 7 we extended the study by testing this protocol in twenty isolates from COLTRYP that were previously identified as TcII by a combination of PCR-RFLP of HSP60, GPI loci and 24S αDNA AFLP markers [33]–[34] and GPI sequencing [32]. We then sequenced these two clones in addition to five TcII isolates with patterns described by Rozas et al. [31] and five isolates exhibiting a similar c5 and c7 pattern (Table 2). The analysis of this locus also allowed examination of a possible phylogenetic significance of this difference. The amplicons (1290 bp) were purified using the Illustra GFX PCR DNA and Gel Band Purification Kit (GE Healthcare Life Sciences, Little Chalfont, Buckinghamshire, UK) and subjected to cycle sequencing reactions with the Big Dye Terminator v 3.1 commercial kit (Applied Biosystems, Foster City, California, USA). The products were sequenced in a 3100 automatic sequencer (Applied Biosystems) using the same primers used for amplification.

**Table 2:** *Trypanosoma cruzi* II isolates subjected to gp72 gene sequencing.

*T. cruzi* II samples	Host species	Municipality-State^a^/Biome	PCR-RFLPgp72 geneprofile^b^	GenBankaccessionnumber
MLD 564b	*Leontopithecus rosalia*	Silva Jardim-RJ/Atlantic Forest	A	KJ402453
MLD 832	*Leontopithecus rosalia*	Silva Jardim-RJ/Atlantic Forest	A	KJ402451
MLD 840	*Leontopithecus rosalia*	Silva Jardim-RJ/Atlantic Forest	A	KJ402452
MLCD 92	*Leontopithecus chrysomelas*	Uma-BA/Atlantic Forest	A	KJ402448
JCA3	*Triatoma brasiliensis*	João Costa-PI/Caatinga	A	KJ402446
LBT 1458 clone 5	*Rhodnius pictipes*	Abaetetuba-PA/Amazon	B	KJ402454
LBT 1458 clone 7	*Rhodnius pictipes*	Abaetetuba-PA/Amazon	B	KJ402455
MLD 594b	*Leontopithecus rosalia*	Silva Jardim-RJ/Atlantic Forest	B	KJ402450
CD 621	*Canis lupus familiaris*	São Roque de Minas-MG/Cerrado	B	KJ402444
CD 640	*Canis lupus familiaris*	São Roque de Minas-MG/Cerrado	B	KJ402445
MLD 1025	*Leontopithecus rosalia*	Silva Jardim-RJ/Atlantic Forest	B	KJ402449
MLCD 82	*Leontopithecus chrysomelas*	Uma-BA/Atlantic Forest	B	KJ402447

a RJ - Rio de Janeiro; BA - Bahia; PI - Piauí; PA - Pará; MG - Minas Gerais;

b A - according to profile described by Rozas et al. [31]; B - profile that was distinct form that described by Rozas et al. [31].

### Sequence analysis

Sequence editing, alignment and phylogenetic tree construction were performed using Chromas v. 1.45 (School of Health Sciences, Griffith University, Queensland, Australia) and the Mega v 5.1 free program [35]. We used the Kimura-2-parameter model and the neighbor-joining statistical methods for phylogenetic reconstruction. The bootstrap was acquired from 1,000 replicate trees. We included analysis of six sequences from GenBank and three TcI isolates as the outgroup in this phylogenetic reconstruction (Table 2). The BLAST (Basic Local Alignment Search Tool) option Nucleotide was used to compare sequences acquired from canine serum with mini-exon *T. cruzi* sequences from GenBank.

### Ethics statement

All dog manipulation procedures were performed in accordance with COBEA (Brazilian College of Animal Experimentation) following the guidelines of the Animal Ethics Committee (CEUA) protocol of FIOCRUZ (Oswaldo Cruz Institute Foundation), Ministry of Health, Brazil. All procedures followed protocols approved by the FIOCRUZ Committee of Bioethics (license 0015-07). In all cases, written consent from the dog owners was obtained, and the owners who also helped us to handle the animals during sampling to avoid incidents. Blood was collected from dogs in non-heparinized vacutainer tubes by puncture of the cephalic vein. No specific permissions were required for conducting this study in the aforementioned locations. We did not manipulate endangered or protected species during our field study.

## Results

### *T. cruzi* II and V/VI in the Amazon region

**Figure 3:**
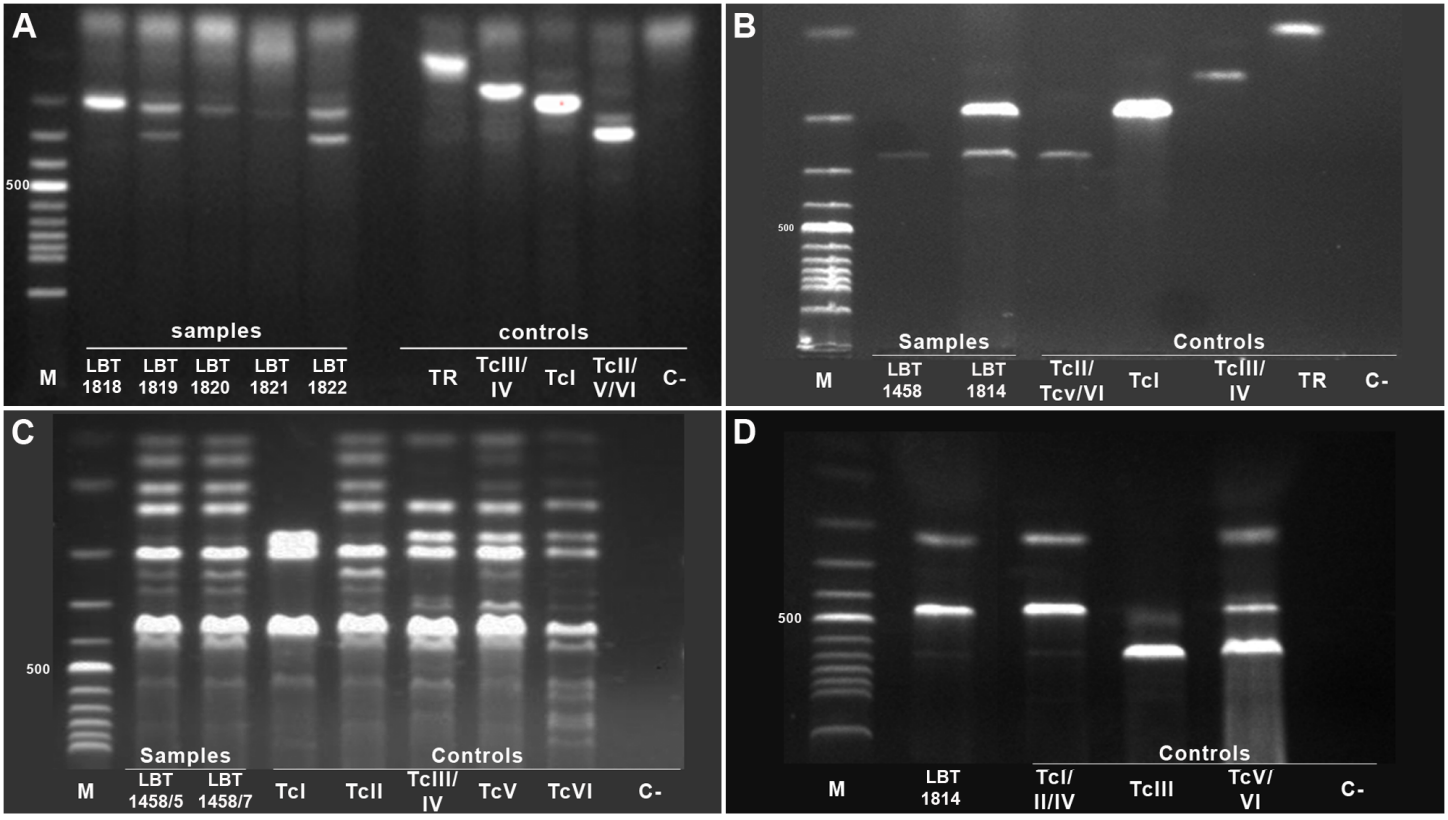
*Trypanosoma cruzi* genotyping from naturally infected dogs and triatomines from the Amazon Biome. (A) PCR product size polymorphisms of the non-transcribed intergenic region of the SL-RNA mini-exon (mini-exon assay) of *T. cruzi* DNA from dog sera from Monte Alegre in the state of Pará, samples: 1- dog LBT 1818, 2- dog LBT 1819, 3- dog LBT 1820, 4- dog LBT 1821, and 5- dog LBT 1822 (B) Mini-exon assay of *Rhodnius pictipes* isolates from Abaetetuba (LBT 1458) and Belém (LBT 1814) in the state of Pará, Samples: 1- LBT 1458 and 2- LBT 1814 (C) *T. cruzi* genotyping profiles for PCR-RFLP with the 1f8 gene/Alw21I restriction enzyme of LBT 1458 clones 5 and 7, Samples: 1- LBT 1458 clone 5 and 2- LBT 1458 clone 7 (D) *T. cruzi* genotyping profiles for PCR-RFLP with histone 3/AluI restriction enzyme for the LBT 1814 isolate: Sample 1- LBT 1814 isolate. The *T. cruzi* DTUs, *T. rangeli* (H-14) and negative controls are indicated in the figure. DTU reference strains: I - Sylvio X/10 cl 1; II – Esmeraldo cl3; III – M5631 cl5; IV–92122102R; V – SC43 cl1; and VI – CL Brener. Agarose gel 3%, stained with ethidium bromide.

The characterization of the samples for *T. cruzi* is summarized in Table 1 and Fig. 3, demonstrating the occurrence of TcII and a Tc hybrid (TcV or TcVI) in the Amazon region in two *Rhodnius pictipes* (respectively LBT 1458 and LBT 1814) and one dog (Fig. 3C, D and Table 1). Another dog (LBT 1819) was found to be infected with Tc2 (TcII/TcV/TcVI) in the mini-exon assay, but due to an insufficient amount of DNA, we were unable to perform additional assays. This is the first report of these genotypes in the Amazonian region.

These three samples were in a mixed infection with DTU TcI as demonstrated by the mini-exon assay. This fact was observed in isolate LBT 1458: the first amplification of the mini-exon gene presented a TcI/Tc2 mixed infection, and in subsequent amplifications of DNA derived from a new extraction, there was only a Tc2 (TcII/TcV/TcVI) profile (Fig. 3B). After cloning, we typed ten of the obtained biological clones and observed that three of them were TcI and seven were Tc2 (TcII/TcV/TcVI) (Fig. 3C). Nevertheless, the PCR-RFLP of the gp72 gene did not allow the characterization of LBT 1458 clones 5 and 7. The identification of TcII in the mixed isolate LBT 1814 was possible using the H3/AluI RFLP protocol (Fig. 3D). We obtained the sequence for the 250 bp fragment (of the mini-exon Tc2 (TcII/TcV/TcVI) group) from the LBT 1822 dog sample and then subjected it to BLAST analyses from NCBI. The sequence obtained showed 100% coverage and 99% identity with three hybrid strains (TcV- SC43 and MN and TcVI - CL Brener). Only 20 to 40% coverage and 100% similarity were observed among the five TcII sequences, although one single TcII strain (Tu18) presented 100% coverage and 99% identity. The profile exhibited by RFLP of the H3 gene using AluI as a restriction enzyme confirmed the hybrid genotype (TcV/TcVI) in dog LBT 1822, but the intensity of the bands was very weak (data not shown).

### Gp72 locus diversity

**Figure 4:**
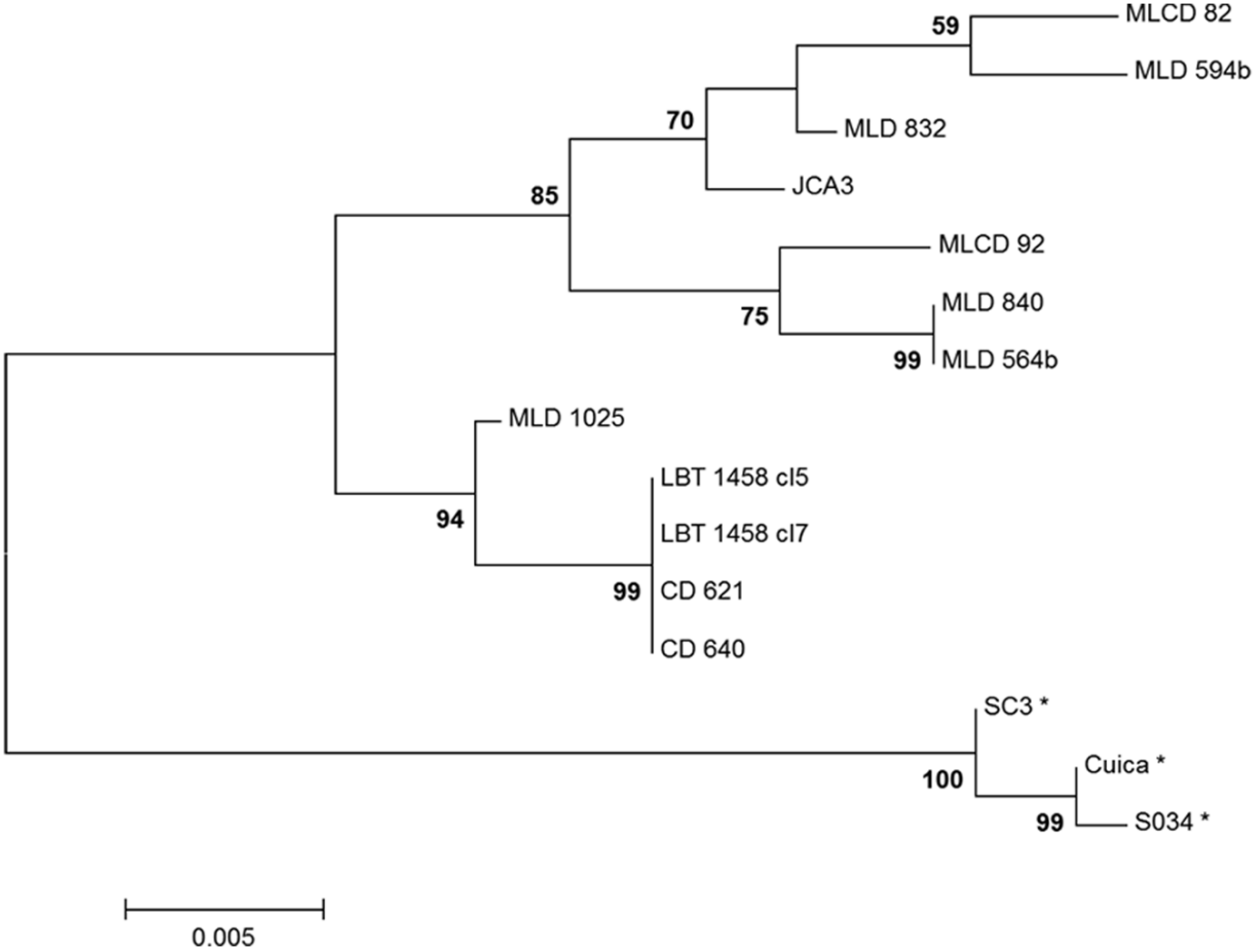
Phylogenetic relationships between *Trypanosoma cruzi* II sylvatic isolates and TcII clones assessed using the gp 72 gene. The tree was constructed using the neighbor-joining method with Kimura-2-parameter distances. Bootstrap values are shown above major clades. MLD: *Leontopithecus rosalia* from Poço das Antas Biologic Reserve in the Atlantic Forest of the state of Rio de Janeiro: MLCD: *L. chrysomelas* from the Una Biologic Reserve in the Atlantic Forest of the state of Bahia, CD: dogs from the Cerrado in the state of Minas Gerais, LBT: *Rhodnius pictipes* from the Amazon Biome and isolate JCA3 is from a *Triatoma brasiliensis* from the state of Piauí in the Caatinga Biome. *GenBank sequences of strain TcI first published by Flores-López et al. [9].

Our TcII isolates were genetically diverse, as revealed by sequencing of gp72 (Fig. 4). The topology of the generated neighbor-joining tree shows the lack of correlation between TcII isolates, considering both geographic and genetic distances (Table 2, Fig. 4). The sequencing analysis showed even more diversity within TcII than was previously observed, highlighting the two patterns of isolates. The two distinct patterns of the gp72 locus by RFLP analysis are due to an SNP (single nucleotide polymorphism) in one of the restriction sites for the enzyme TaqI. Isolates with a thymine to guanine mutation are not cleaved by the enzyme.

## Discussion and Conclusion

Trypanosomiasis caused by *T. cruzi* is a zoonosis that is widely dispersed in nature in the Americas. Due to a the large increase in the occurrence of this parasite difficulties involving the capture and transport of biological materials and parasite isolation from wild mammals and vectors, data on the distribution of *T. cruzi* genotypes are aggregated and do not reflect all habitats where this parasite occurs. The greatest barrier to understanding the transmission cycle ecology of *T. cruzi* DTUs is the challenge inherent to fieldwork. As a consequence, there are still numerous areas and mammal species that have been inadequately or never sampled. Additionally, only wild animal isolates from blood culture or xenodiagnosis are typed, and mammals with subpatent infections based on positive serological tests are never considered. Therefore, an important bias is introduced that certainly results in misinterpretations of alleged associations between genotypes of *T. cruzi* with host species. In addition, the peculiarities of interactions among each subpopulation (potentially different DTUs) with their respective hosts may produce distinct selective pressures that result in higher or lower success in the isolation of parasites through the current available methods. This bias may result in misinterpretation of the ecology and biology of this trypanosomatid. The Amazon region biome has especially attracted our attention in recent years due to an increase in cases of Chagas disease transmitted by the oral route and the scarcity of data concerning the ecology of *T. cruzi* genotypes in this large and diverse biome [2], [3], [22]. The apparent difference in the prevalence of the *T. cruzi* DTUs in nature is intriguing and may be influenced by the different patterns of infection among each *T. cruzi* DTU and its mammalian hosts. Although these strategic differences have not yet been fully clarified, all *T. cruzi* DTUs were found to be successful because of their maintenance in nature. Most likely, each DTU establishes a particular interaction pattern with their several host species. After a short period of high TcII parasitemia, which is detectable in hemocultures, for example, we observed that although the hosts remained serologically positive, it was no longer possible to recover the parasites by hemoculture [21]. This suggests that the transmission strength of these animals is restricted to a short period of approximately two months [21]. Opossums (*Didelphis aurita*) handle TcII in a different manner: in experimental infections with Y and FL strains, both of which are TcII, these mammals were able to control parasitemia even when very young and still dependent on the marsupium [36]. Nevertheless, naturally infected *Didelphis* spp are able to maintain TcII in the wild. In contrast, the four-eyed opossum (*Philander frenata*) was able to maintain long-lasting infections with a high prevalence of positive hemocultures when experimentally infected with the Y strain (TcII) [37]. A long-lasting TcII transmission cycle in the wild has been described in two tamarin species, *Leontopithecus rosalia* and *L. chrysomelas*, in two fragments of the Atlantic Forest Biome [38]. Additionally, the carnivore *Nasua nasua* in the Pantanal biome was demonstrated to be a suitable wild TcII host [13].

In this study, we show for the first time the presence of *T. cruzi* DTU TcII and one hybrid DTU (TcV or TcVI) in the state of Pará in the Brazilian Amazon. These data together with previous data from our group show that TcII is present in wild transmission cycles in all Brazilian biomes (Fig. 1) and is not restricted to areas below the Amazonian Basin, as has always been assumed [2], [3]. Additionally, the presence of infected triatomine bugs and dogs by TcII and hybrid DTUs imply that other mammals and triatomine bugs should also be infected and involved in the transmission cycle of these DTUs in Pará. Notably, before this study, a hybrid DTU was only reported in wild mammals in Brazil once, in a sylvatic rodent species, *Thrichomys laurentius,* in the northeastern region of the country [17], demonstrating that we are still far from understanding the distribution and hosts of hybrid DTUs. The report of a Tc hybrid in the Amazon leads to even more questions, mainly due to the large gap in reports of these DTUs in Brazil, as most of the isolates that we obtained were from the southern part of the country and derived from humans, and only one isolate was reported in a wild rodent from the northeastern Caatinga [17].

The characterization of *T. cruzi* by multiplex PCR fo the mini-exon gene, developed by Fernandes et al. [29] and validated by Aliaga et al. [39], was employed as the first step to type *T. cruzi* DTUs. This method allows a reliable characterization of TcI and distinguishes two DTU groups, TcIII/TcIV and TcII/TcV/TcVI. It is a valuable method because *T. cruzi* isolates derived from the wild environment very often include more than one DTU. Due to the nature and the peculiarities of the samples, it became necessary to use different typing techniques. Therefore, because serum samples that contain small quantities of *T. cruzi* DNA consequently result in low DNA recovery rates, our technique of choice was multiplex PCR of the mini-exon gene because this gene occurs in multiple copies per cell (approximately 200),thereby increasing our chances of success. Moreover, all attempts to type this material using other targets, such as H3, were unsuccessful because these are single copy genes. We used the same rationale employed for assays using ancient DNA – using multi-copy genes [1], [40]–[41]. The presence of both TcI and Tc2 (TcII/TcV/TcVI) fragments in the serum samples led us to perform molecular cloning of the amplicons to select and subsequently sequence these TcII clones. Another rationale for this technique is that the same parasite may contain minor nucleotide variations, which results in double peaks in the chromatogram. Obtaining a single sequence allows a comparison between gene sequences that are already deposited in the gene bank.

A different situation was presented by isolates 1814 and 1458, which were derived from *R. pictipes* that displayed mixed TcI and Tc2 profiles, respectively, when typed using the mini-exon gene. Once the cultures were available, the amount of DNA was not a limiting factor, and it was possible to type the DTUs using single-copy genes such as H3 and 1f8. The decision to perform biological cloning was made because the first typing using the mini-exon gene showed a mixed profile. Later typings of the same material, however, revealed only Tc2. Biological cloning has allowed us to choose and use 1f8/Alw21I to type 1458 because this target displays a unique profile for TcII, as shown in Fig. 3C. In the case of isolate 1814, biological cloning did not appear to be necessary because we had no conflicting results when we re-characterized the material as in the previous case. Thus, we were certain that this was an isolate containing TcI and Tc2. This allowed us to choose another option for typing mixed TcI/TcII isolates, which was to eliminate the possibility of dealing with the hybrid genotypes (TcV and TcVI). In the latter case, we used the same method described by Rocha et al. [21].

The isolation of TcII from *R. pictipes* demonstrates the ability of this triatomine species to maintain this genotype, in contrast with previous reports. Moreover, these studies were conducted in experimentally infected *Rhodnius prolixus* [42]–[44] and were based on observations of only three TcII strains (Y, AF-1, Tu18), which do not represent the diversity of this genotype in the wild. Although natural infections by *T. cruzi* in *Rhodnius* sp in the Amazonian biome have been studied intensely over the past few decades, the majority of these studies did not employ molecular genotyping tools [22]. Studies of natural infection with *T. cruzi* in *R. pictipes* in the Amazon region that aimed to characterize the parasite isolates have reported infections only with TcI, TcIV and *T. rangeli* [24], [45].

The greater issue challenging our understanding of the populational structure of the TcII DTU and hybrids is their apparent focal distribution in nature. Among the inherent difficulties of detecting and characterizing mixed infections is the selective pressure exercised by axenic medium. An insufficient understanding of the behavior and dynamics of TcII and Tc hybrid infection in mammals and vector species is likely the consequence of selective pressures on the process of isolation and maintenance and different sensitivities of in diagnostic methods, in addition to collection bias. This was clearly demonstrated in the diagnosis by PCR of a TcII infection in the serum samples of three specimens of *Didelphis aurita* from an outbreak area in the state of Santa Catarina (Maldonado et al., unpublished data), whose hemoculture was characterized as TcI [25]. Another example is a *T. cruzi* isolate obtained from *Didelphis albiventris* from the Caatinga biome that after several years maintenance in the COLTRYP of our laboratory was always characterized as a single TcI infection by different typing techniques [29], [31] but was then demonstrated to also include TcII after biological cloning was performed (Lima et al., unpublished data). The same event was observed in this study with isolate LBT 1458, which was characterized as mixed TcI/Tc2 in the first mini-exon assays and in a second DNA extraction, demonstrated only the Tc2 (TcII/TcV/TcVI) band pattern (Fig. 3B). Moreover, the presence of TcI in this isolate was subsequently demonstrated by biological cloning. These factors contribute to a misinterpretation of the prevalence of *T. cruzi* DTUs in nature. Additionally, the large number of sylvatic mammals with positive serology that do not result in recovery of parasites by hemoculture (70%) may also contribute to an underestimation of parasite prevalence.

The RFLP of the gp72 gene discriminated two groups among the Brazilian TcII isolates, one with a previously known profile and another with an unusual profile. The sequencing of this locus points to an SNP that is responsible for these two profiles, although a phylogenetic reconstruction showed that the presence of the same SNP does not imply higher phylogenetic relatedness. The genetic diversity among twelve sylvatic isolates and clones from Caatinga, Cerrado, Atlantic Forest and Amazon (Fig. 4) in a constitutive gene reinforces our hypothesis that TcII has a broader distribution and higher prevalence of infection than is currently reported because a common assumption of such an analysis is that diversity is directly related to the size of a given population [46]–[47]. The absence of a correlation between geographic area and the two TcII sequencing patterns suggests that variants of TcII are also most likely not limited to a group, such as the tamarin species of the Atlantic Forest, but include other mammalian host species in other biomes [35].

Classically the TcII DTU has been associated with the intradomiciliar transmission of *T. cruzi* by *Triatoma infestans* and with severe cases of Chagas disease [1], [3]. Indeed, this was the epidemiological profile that the Southern Cone initiative successfully targeted and controlled, leading to a decrease in the importance of this epidemiological scenario. Additionally, the TcII DTU was classically described as being prevalent in the region just below the Amazon, specifically the Midwest region of Brazil. Moreover, in recent years, increasingly robust evidence of a wide distribution of TcII in free-living wild animals has emerged [13], [37]–[38] Our present finding of TcII in Amazonia refutes two paradigms: (i) the association of this DTU with a particular biome and host species and (ii) TcII association with human infection and even severe Chagas disease because no single human case of TcII infection in Amazonia has been detected. Altogether, more than 100 years after the description of *T. cruzi* by Carlos Chagas, who even then noted the diversity of this taxon [48], the scientific community still cannot answer one of the first questions asked by that brilliant scientist: “What do the different forms of the parasite suggest?” We dare say that we are still far from deciphering this mystery.
